# Evaluating the Adipose Tissue Depth as a Predictor Factor for Gestational Diabetes in Later Pregnancy—A Systematic Review

**DOI:** 10.3390/biomedicines11051492

**Published:** 2023-05-22

**Authors:** Bianca-Margareta Salmen, Valeria-Anca Pietrosel, Cristiana-Elena Durdu, Teodor Salmen, Cosmina Theodora Diaconu, Ioana-Cristina Bica, Claudia Gabriela Potcovaru, Florentina Gherghiceanu, Roxana-Adriana Stoica, Anca Pantea Stoian

**Affiliations:** 1 Doctoral School, “Carol Davila” University of Medicine and Pharmacy, 020021 Bucharest, Romania; 2 Department of Diabetes, Nutrition and Metabolic Diseases, “Prof. Dr N.C.Paulescu” National Institute of Diabetes, Nutrition and Metabolic Diseases, 030167 Bucharest, Romania; 3 Department of Obstetrics and Gynecology, Filantropia Hospital, 011171 Bucharest, Romania; 4 Department of Diabetes, Nutrition and Metabolic Diseases, Carol Davila University of Medicine and Pharmacy, 050474 Bucharest, Romania

**Keywords:** gestational diabetes mellitus, adipose tissue depth, pregnancy, ultrasound

## Abstract

The increasing prevalence of gestational diabetes mellitus (GDM) requires non-invasive and precise techniques for evaluating the predisposing risk factors such as visceral adipose tissue (VAT) and subcutaneous adipose tissue (SAT). According to PRISMA, we developed a systematic review and searched after “visceral adipose tissue AND gestational diabetes” and identified 221 articles on the MEDLINE and Word of Science databases. After assessing them for inclusion criteria and two researchers screened them, 11 relevant articles were included. Although evidence is conflicting, more studies favor using US-determined VAT in GDM prediction. VAT may be more valuable than body mass index or SAT in predicting GDM. VAT can represent an additive factor to the prediction tool of the risk of developing GDM when used in conjunction with other anthropometric or biological parameters or maternal risk factors. US measurements are heterogeneous given different evaluation techniques, cut-off values and inter-operator variation. A significant limitation is the lack of a gold standard to identify GDM confidently. Pregnant women may benefit from early monitoring and preventive care if classified as high risk for GDM early in the gestational period. US-measured VAT during the first trimester of pregnancy seems a valuable and inexpensive screening approach to predict GDM development later in pregnancy, either by itself or if used in conjunction with other clinical and biological parameters.

## 1. Introduction

Gestational Diabetes Mellitus (GDM) complicates 16% of pregnancies, with its prevalence worldwide ranging between 2–38%, while the prevalence reported in the United States was 7.8% in 2020, with a generally positive trend [1,2,3]. GDM is defined by The American Diabetes Association (ADA) as diabetes diagnosed in the second or third trimester of pregnancy that was not clearly overt diabetes prior to gestation [4].

Although several approaches to GDM screening and diagnostic criteria have been proposed over the years, there has yet to be a universally accepted standard procedure [4,5,6], all methods having their limits and advantages. The consensus statement from the International Association of Diabetes and Pregnancy Study Groups (IADPSG) suggests using the 75 g oral OGTT as both the screening and the confirmatory test at 24 to 28 weeks of gestation among all pregnant women not previously diagnosed with DM by random or fasting plasma glucose testing at the first antenatal visit. The blood glucose cut-off levels used by the IADPSG criteria were arbitrarily designated as those that increase the risk of DM-induced adverse pregnancy outcomes by a 1.75-fold, rather than endpoints such as prediction of subsequent maternal DM [6]. Using the IADPSG criteria, GDM prevalence has significantly increased [4,5] in parallel with the obesity epidemic and older maternal age [4,7,8]. IADPSG, ADA, World Health Organization (WHO) and other societies also support screening for early abnormal glucose metabolism at the first antenatal visit using standard criteria, especially in women with risk factors for GDM or those who did not benefit from a DM screening prior to pregnancy, although there is no consensus on the optimal diagnostic and therapeutic management for this group [4,9,10].

The most important risk factors are represented by a family history of diabetes mellitus (DM), especially when present at a first-degree relative, maternal age ≥35 years, body mass index (BMI) ≥30 kg/m^2^, history of impaired glucose tolerance (IGT), impaired fasting glucose (IFG) or A1c haemoglobin ≥5.7%, personal history of a prior birth of a more than 4000 g infant or personal history of GDM in a precedent pregnancy, conditions associated with insulin resistance (IR) (e.g., polycystic ovary syndrome), certain groups (Alaska natives, Hispanic Americans, South or East Asians and Native Americans) [11,12,13,14,15,16]. GDM is a complex disorder as it affects both the mother by increasing the risk of preeclampsia, birth injuries, cesarean delivery rates, the risk of further developing type 2 DM (T2DM) within five years after birth, and the fetus by an increment of the risk of developing neonatal hypoglycemia, hyperbilirubinemia, polycythemia, hypocalcemia, macrosomia rate or newborn birth trauma or perinatal mortality [9,17,18]. In addition, GDM is a risk factor for adverse pregnancy outcomes as well as it is described in Rheumatoid Arthritis pregnancies [19,20].

As the incidence of GDM is exponentially rising, there is a need to identify pregnant women at risk of developing GDM. In this direction, there is constant research to establish early markers for further development of GDM to offer nutritional counseling and optimum care to prevent or reduce the maternal–fetal effects of GDM. There are described clinical parameters, biological markers and ultrasonographic (US) measurements to predict GDM. Clinical parameters are represented by high BMI and waist-to-hip ratio (WHR). A meta-analysis published in 2008 by Torloni et al. concluded that the pre-pregnancy BMI is linearly associated with the risk of developing GDM, as underweight women presented an odds ratio (OR) of 0.75 (95% confidence interval (CI) 0.69 to 0.82), overweight patients presented an OR of 1.97 (95% CI 1.77–2.19), while patients with morbid obesity had an OR of 5.55 (95% CI 4.27–7.21). Another impressive finding by Torloni et al. was that every 1 kg/m^2^ added to the initial BMI augmented the risk of GDM by 0.92% (95% CI 0.73–1.1) [21]. WHR represents a clinical measurement, appearing to be a promising method of predicting the risk of developing GDM among pregnant patients; the usefulness of WHR in pregnant women is currently debated, being necessary more studies in this direction, as the present evidence is controversial [22,23,24].

Although BMI is the conventional measure used to define obesity, it has some limitations, mainly driven by its inability to reflect body composition, especially during pregnancy. Waist circumference (WC) better depicts central adiposity and is associated with obesity-related comorbidities but does not distinguish between subcutaneous adipose tissue (SAT) and visceral adipose tissue (VAT) and has limited accuracy in pregnancy as uterine growth alters the abdominal compartment. In addition, precise imagistic techniques such as bioelectrical impedance (BEI), dual-energy X-ray absorptiometry and computerized tomography (CT)-described as the gold standard, widely used in the general population to measure visceral fat thickness precisely, are expensive and cannot be used in pregnancy given the redistribution of body water and the exposure of the fetus to radiation [25,26,27].

US measurement of the VAT and SAT thickness in the first or second trimester has gained popularity lately as it is non-invasive, inexpensive and easy to perform, especially during the first and second-trimester anomaly screening. In addition, it has the advantage of being a validated technique, with a strong correlation with CT-measured VAT, excellent inter-observer coefficients of reliability, excellent reproducibility and repeatability [27,28,29], but there are different opinions about its predictive ability of GDM in the current literature [29,30,31]. As there is no consensus concerning the role of VAT and SAT measurements, we conducted a systematic literature review and evaluated studies including SAT and VAT as potential predictors for developing GDM.

## 2. Materials and Methods

**Figure 1:**
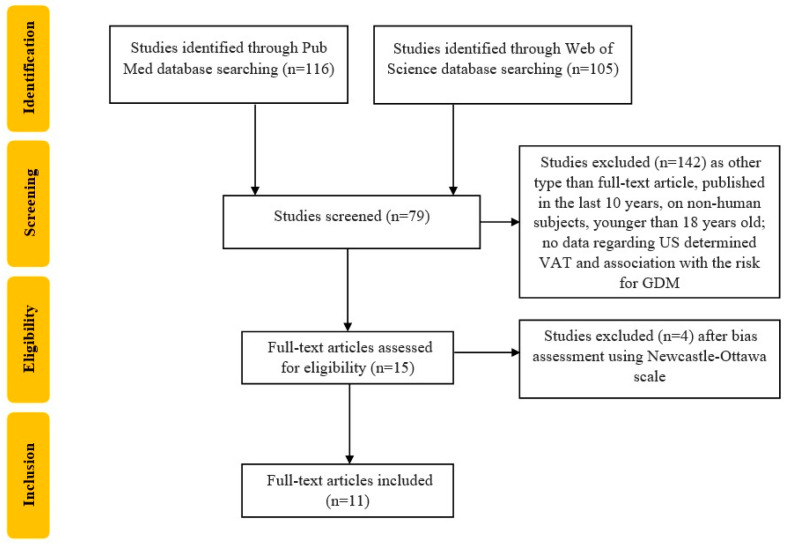
Flowchart of the study selection process according to PRISMA recommendation. US—ultrasound; VAT—visceral adipose tissue; GDM—gestational diabetes mellitus. The present article analyzed data from each article, focusing on the association between US-determined VAT (and SAT or TAT if available) and risk for GDM development measured as odds ratio (OR), risk ratio (RR), CI (confidence interval) or *p*-value depending on the way the study reported its results. We provided a narrative synthesis, using text and tables in order to describe the summary and explanation of the study characteristics and findings. If present, further comparison of the prediction power of VAT or SAT with that of other anthropometric parameters such as BMI, WHR, WC, etc., will be evaluated and described in Section 4.

We developed an easily reproducible protocol for our study following the recommendations of Preferred Reporting Items for Systematic Reviews and Meta-Analyses (PRISMA) for the systematic review protocol checklist, registered with CRD42023389055 number. Furthermore, we used the Population, Intervention, Comparison, Outcome and Study Design (PICOS) strategy to guide our study rationale and to make a clear, useful and systematic literature search. First, we searched using the following criterion: “adipose visceral tissue AND gestational diabetes” and identified 221 articles (115 on the MEDLINE database and 106 on the Web of Science database, respectively), until 28 February 2023. We also performed a manual search of the references to identify other potentially useful articles missed by our search strategy. After assessing that the studies are only full-text original articles published in English, in the last ten years and only on the adult human population (age over 18 years old), 79 studies were identified. We included studies with US evaluation of VAT in normoglycemic pregnant women in 1st or 2nd trimester with an OGTT at 24–28 weeks for GDM diagnosis or dysglycemia. Articles that only assessed SAT, without data about VAT, were excluded, as well as studies including women with known DM. Two researchers individually performed the screening in order to find relevant articles to our theme of interest and if any disagreements occurred in the selection process, they were settled down by a third reviewer. After bias assessment using the Newcastle–Ottawa Scale [32], eleven articles were included, as shown in Figure 1.

## 3. Results

For a better synthesis and understanding, the results are shown in Table 1 and Table 2. 

For each study, the year of publication, country and number of participants were reported alongside with parameters such as GW of assessment, type of US technique used, the GDM incidence and other relevant anthropometric findings were included and reported in Table 1. 

The maternal age, anthropometric parameters, the mean values for VAT, SAT, TAT and other US-determined parameters if reported alongside with their correlation with GDM occurrence, disglycemia during pregnancy or other relevant metabolic disturbances were synthesized in Table 2.

## 4. Discussion

The use of new tools for early GDM screening combining safety, low cost and high efficacy can contribute to identifying pregnant women at high risk for glycemic disturbances and can trigger strategies to achieve metabolic control early during pregnancy as a method of reducing maternal–fetal risks [23] and a better allocation of financial resources in this high-risk population [25]. Furthermore, if the US determination of adipose tissue correlates with other anthropometric indices and can reliably predict GDM from early pregnancy and allow a skip from OGTT later in low-risk pregnancies has to be further evaluated, given the high variability for the VAT thresholds. All of the aforementioned studies supported the utility of US-determined VAT in identifying high-risk pregnancies as greater VAT was associated to increased risk for GDM or disglycemia later in pregnancy [7,25,28,29,30,33,34,35,36,37,38,39]. Martin et al. first described the association between visceral adiposity in early pregnancy and glucose intolerance in later pregnancy in 2009. In 62 pregnant women, they observed that a VAT above the upper quartile value was associated with an increased risk for a positive glucose challenge test between 24–28 weeks of gestation [40]. Most of the included studies have shown that US-measured VAT in an earlier pregnancy may predict glucose intolerance, IR and GDM later in pregnancy and can be correlated with metabolic syndrome (MetS) features in early pregnancy. A recent comprehensive meta-analysis of Rahnemaei et al. evaluating the use of body composition indices in the early stages of pregnancy in predicting GDM also confirmed that VAT and SAT are associated with risk for GDM (although the values were higher in the GDM group, the difference for SAT was not statistically significant) as well as other markers such as neck circumference, WC, hip circumference, WHR, arm circumference and short stature [41]. Another meta-analysis showed a direct relationship with GDM of indices of general body obesity including VAT, WC and WHR [42].

Regarding other screening strategies, Thaware et al. showed that US-measured VAT ≥ 42.7 mm in early pregnancy compared with current UK’s National Institute of Health and Care Excellence (NICE) criteria—screening at 24–28 gestational weeks only for women with specified risk factors—had greater sensitivity (Sen) (87% vs. 40%, respectively; *p =* 0.02) and similar specificity (Spe) (62% vs. 74%, respectively; *p =* 0.15) for identifying GDM and, as the authors mentioned, it could reduce by half the number of pregnant women requiring an OGTT screening as per NICE criteria [28].

### 4.1. Used Techniques

Several methods of US measurement of adipose tissue have been proposed. For instance, Rocha et al., Bourdages et al. and Alves et al. used the technique by Armellini et al. with or without slight modifications as described by Martin et al.: VAT was measured as the perpendicular distance between the posterior aspect of the junction of the two rectus abdominis muscles (i.e., the linea alba) and the anterior aspect of the abdominal aorta; measurements were made in the supine position, and the probe was placed on the anterior abdomen in the xipho-umbilical line, 1 cm above the umbilicus [7,25,30,43,44]. D’Ambrosi et al. used a technique introduced by Suzuki et al. [45] in non-pregnant women, and the US index showed a high correlation with CT measurements of VAT and SAT [29]. Accordingly, SAT was measured as the maximum vertical distance from the skin line to the anterior edge of the linea alba, immediately caudal to the xiphoidal tip. In contrast, VAT was measured on the same image from the posterior edge of the linea alba to the anterior surface of the liver.

Furthermore, the authors state that this method can be applied at any gestational age since the measurement performed at the level of the xiphoid process is not affected by the increase in uterine volume during the different trimesters of pregnancy [29]. In Gur et al. study, VAT was defined as the fat thickness between the liver surface and the linea alba [34]. Irrespective of the chosen method, techniques had to be validated after proving their high reproducibility. If the assessment was performed by a single ultrasonographer or two individuals, ensuring good reliability is also important. A recent study found that US measurement at 12 weeks of gestation can produce reliable, repeatable and accurate measures of AT during pregnancy, with acceptable intra-observer precision for measures of SAT, VAT and TAT according to the anthropometric criterion, with higher precision reported in SAT values than in VAT. Inter-observer reliability assessed by Limits-Of-Agreement (LoA) confirm anthropometrically reliable to 0.5 cm. Systematic bias was minimal for both measures, falling within 95% CI [45].

### 4.2. Different Thresholds for Predictive Value

Once the association of VAT depth with GDM was confirmed, at further statistical analysis, a cut-off value with the best predicting value for GDM was searched. For instance, Thaware et al. reported that a VAT depth ≥42.7 mm had a Sen of 87% (95% CI 60–98%) and 62% Spe in the early third trimester [28]. De Souza et al., in a more extensive observational study (*n =* 485), found that an elevated VAT depth (>48 mm) at 11–14 weeks gestation was associated with both dysglycaemia and GDM (according to IADPSG criteria), with ORs of 3.1 (95% CI 1.1–9.5) and 3.4 (95% CI 1.5–8.0), respectively, after adjustment for maternal age, ethnicity, family history of DM and BMI [33]. Rocha et al. divided the study group based on pre-gravid BMI into obese, overweight and non-obese. They observed that according to the ROC curve, a 45 mm threshold was the best cut-off value, with 66% of accuracy in predicting GDM (crude and adjusted OR of 13.4 (95% CI 2.9–61.1) and 8.9 (95% CI 1.9–42.2)). However, among pre-gravid obese patients, this threshold did not reach statistical significance to predict GDM. For this category, additional studies are needed to determine the best VAT cut-off to ensure low GDM risk later in pregnancy [25]. In 2009, Martin et al. found that VAT depth >47.4 mm was associated with an increased risk for an OGTT ≥7.8 mmol/L at 24–28 weeks’ gestation OR of 17.3 (95% CI 18–163.8) before adjustment and 16.9 (95% CI 1.5–194.6) after adjusting for maternal age and pre-gravid BMI [40]. Gur et al. reported that the optimal cut-off points for predicting GDM were VAT 19.5 mm [(AUC) = 0.66, *p =* 0.043], WC 103.5 cm (AUC = 0.64, *p =* 0.079) and BMI 34.5 (AUC = 0.64, *p =* 0.069) [34]. Last but not least, Alves et al. showed that the optimal VAT cut-off for maximized Youden’s index was 5.1 cm, and a 1 cm increase in VAT led to unadjusted OR for developing GDM of 1.99 (95% CI 1.59–2.46) and after adjusting for maternal BMI and age, the OR was two (95% CI 1.61–2.5) [7].

### 4.3. Is Pre-Pregnancy BMI Better Than VAT in the Detection of GDM?

VAT measurement may be superior to BMI in predicting GDM, as BMI does not reflect the metabolically active AT to the same degree as VAT [7,41]. A fierce debate about whether VAT or SAT has better predictive value than BMI is still ongoing, with literature offering mixed results. Furthermore, although there are data supporting an additive role of VAT to BMI in predicting GDM [26,29,30,35], it is still questioned whether US-measured VAT offers improved discrimination in detecting GDM compared with simply using pre-pregnancy BMI. For instance, Alves et al. showed that higher VAT might better predict GDM than pre-pregnancy BMI, with a higher ROC curve for developing GDM for VAD (0.70, 95% CI 0.63 to 0.75) than for pre-pregnancy BMI (0.57 95% CI 0.50 to 0.64) (*p* < 0.001) [7]. De Souza et al. showed that an elevated VAT, assessed by the US at 11 to 14 weeks gestation, independently predicted the risk of dysglycemia later in pregnancy [33]. In a cohort of 1048 pregnant women, Bourdages et al. observed that first-trimester VAT was associated with a higher possibility of developing GDM, especially insulin-requiring GDM, and when used alone, the discriminative value of first-trimester TAT was similar to that of BMI [30]. However, when TAT, BMI and maternal age were considered together, the detection rate increased to 42% at a 10% false-positive rate. The authors mentioned the low prevalence of GDM in the sample (5.8% and 3.4% for insulin-requiring GDM) as a possible reason for the poor performance of the US. Rocha et al. also concluded that VAT is better than pre-pregnancy BMI in predicting GDM, having a higher Sen (89% vs. 55%) [25]. Analyzing based on pre-gravid BMI, they report that non-obese pre-pregnant women at risk for GDM can be detected using VAT above 45 mm with a Sen of 88%. In contrast, in obese women, a VAT <45 mm can suggest a group with a low risk of GDM, with a negative predictive value of 94%. Suresh et al. also concluded that SAT at 18–22 weeks gestation as a “surrogate” for central obesity is better than BMI as a marker for obesity-related pregnancy outcomes [26]. On the contrary, Aydin et al. showed that although they observed statistically significant differences between the GDM and non-GDM groups in terms of current BMI, SAT and intraperitoneal fat thicknesses and WC and hip circumference values, the logistic regression model showed that only current BMI had a significant association with the increasing GDM frequency. However, the authors mentioned the small sample size as a limitation that could affect the statistical analysis [31]. 

### 4.4. Adipose Tissue and Metabolic Syndrome (MetS) Features

GDM is also related to MetS [34,46]. Although several features of the MetS occur during normal pregnancy such as adipose tissue and IR increase, pregnancy and MetS are not superposable and can affect both mother and offspring [27]. Some studies showed that MetS, present in early pregnancy (as by standard diagnostic criteria), increased the risk for GDM by 2–4 fold; even after adjusting for BMI and individual metabolic markers such as raised triglycerides (TG) or low-density lipoprotein cholesterol or reduced high-density lipoprotein cholesterol (HDL-C) also pose a significant risk for developing GDM [46]. In addition, first-trimester hyperinsulinemia precedes the onset of hyperglycemia in the second trimester of pregnancy [27]. Hence, metabolic markers that can be modified by diet and lifestyle must be assessed, allowing for early detection and management [46].

The association of adipose tissue depth with abnormal glucose homeostasis and other metabolic markers has also been researched. Bartha et al. examined 30 women at 11–14 weeks of gestation and found a significant association between the US measurement of VAT and fasting glycemia (r = 0.37, *p =* 0.04), insulinemia (r = 0.59, *p =* 0.001) and insulin sensitivity as assessed by HOMA-IR index (r = 0.59, *p =* 0.001); however, given the small sample size, the results should be interpreted cautiously [47]. De Souza et al. also concluded that measuring maternal VAT and TAT at the time of routine first-trimester US might provide additional information about maternal IR beyond pre-pregnancy BMI, explaining 42% and 46%, respectively, of the variance in HOMA-IR [48]. The same group, later on, in a prospective cohort study of 485 pregnant women revealed an association between first-trimester VAT (OR 3.1, 95% CI 1.1–9.5) or TAT (OR 2.7, 95% CI 1.1–7.8) and the risk of a combined endpoint of IFG, IGT or GDM [31] and that maternal hepatic fat, VAT and TAT in mid-pregnancy independently predicted GDM and impaired glucose homeostasis [35]. From a broader perspective, Gur et al. showed that VAT maximum value was positively correlated with diastolic blood pressure (*p =* 0.03), TG (*p =* 0.01), FBG (*p =* 0.04), fasting insulin level (*p =* 0.03) and IR assessed by HOMA-IR (*p =* 0.01) and negatively correlated with HDL-C (*p =* 0.04) measured at 4–14 gestational weeks. Furthermore, comparing VAT, SAT, BMI and WC, only VAT maximum value correlated with IR in the HOMA-IR model. The authors mentioned the small sample size which can affect the results however [34]. Another study on 83 Egyptian pregnant women showed that measurement of VAT during a routine 11–14 weeks’ gestation US might improve the performance of screening for GDM and that it correlated with metabolic risk factors even better than BMI. Authors calculated both IR using HOMA-IR model and insulin sensitivity using ISI (insulin sensitivity index) which employs fasting and postprandial plasma glucose and insulin, with a positive relationship between VAT and HOMA-IR and negative with insulin sensitivity. Moreover, this study showed no significant relation between SAT and HOMA-IR, explaining that VAT is more metabolically active than SAT [36]. However, although HOMA-IR model was frequently employed given it is simple, minimally invasive and proved to be a robust clinical and epidemiological tool for the assessment of IR, it mainly indicates predominantly hepatic IR. Therefore, it would be worth studying the correlation of VAT and IR indices derived not only from fasting values but also those derived from OGTT which better indicate the peripheral IR [49] as there were studies that reported marked variability between the IR indices in pregnant women [50]. On the other side, Pontual et al. observed that VAT measured in the first half of pregnancy (15th–20th weeks) was no better than pre-pregnancy BMI, predicting IR (assessed by HOMA-IR) and dyslipidemia later in pregnancy. They proposed three limitations that might explain poor US performance: late inclusion (around the 19th week of pregnancy), technical aspects of US measurement (such as maternal body habitus) and the inability of the authors to exclude women with MetS or pre-existing GDM [27].

VAT is a better reflection of the body fat distribution [51] and is considered the metabolically active compartment of the adipose tissue. It is strongly associated with disease risk, primarily related to increased IR, hypertension and cardiovascular disease [25,26]. In non-pregnant populations, visceral adiposity is an independent predictor of IR, MetS and T2DM [33]. Although more knowledge is needed to understand the underlying pathophysiological ways, several mechanisms are proposed. Due to the anatomical position of the VAT and the capability of draining the contents directly to the liver through the portal circulation, it increases the hepatic inflow of free fatty acids and adipokines, which contribute to inflammation, oxidative stress and by further IR, glucose intolerance and dyslipidemia to increased metabolic risk [25,33,34]. VAT has an altered inflammatory state in GDM women [52,53]. It is considered that VAT is more related to abnormal glucose homeostasis and GDM in pregnancy rather than SAT [29,33,34,53], given its heterogeneous histology, with two distinct deep and superficial layers [54], of which the former exhibits metabolic activity similar to that of VAT [33]. One study conducted by Yang et al. on 333 Korean women with singleton pregnancies in the first trimester reported that SAT was a statistically significant predictor of GDM [55], and a longitudinal cohort study of 1510 pregnant women in Australia suggested the potential of SAT as an independent predictor of GDM [56].

The etiology of GDM is multifactorial [29], with multiple studies incriminating the association with increased adipose tissue, increased pre-pregnancy BMI or excessive gestational weight gain [57]. 

### 4.5. An Exhaustive Formula That Estimates the GDM Risks

Measurement of VAT could represent a useful and inexpensive instrument to evaluate the risk of developing GDM. Its inclusion in the first trimester of pregnancy at the usual first antenatal visit may provide an additional contribution to early identification [26,29,30,35] of women at high risk of developing GDM or other pregnancy complications [29,58], alongside other parameters such as maternal age and BMI. Other anthropometric variables (weight, height, mid-upper arm circumference, circumferences of calf and neck and triceps skinfolds and subscapular skinfolds) were evaluated in a study. Accordingly, the best measurements that correlated with VAT and TAT were mid–upper arm circumference and subscapular skinfolds, both of which showed a higher correlation than pre-pregnancy BMI, and those could represent a low cost, efficient and replicable estimate of VAT and TAT in an outpatient clinic environment, especially in low- and middle-income countries [58]. In addition, measuring AT and BMI can likely increase the value of biochemical and biophysical markers proposed for early pregnancy prediction of GDM [30]. A study by Liu et al. found that US maternal epicardial adipose tissue (EAT) thickness is positively and significantly associated with both the risk of GDM and other adverse outcomes related to GDM. The univariate regression analysis revealed that maternal age (OR = 1.05, 95% CI: 1.01–1.09), BMI (OR = 1.05, 95% CI: 1.01–1.09), TG (OR = 1.23, 95% CI: 1.04–1.46), total cholesterol (OR = 1.21, 95% CI: 1.04–1.41), HDL-C (OR = 0.42, 95% CI: 0.22–0.79), and EAT thickness (OR = 2.92, 95% CI: 2.54–3.36) were significantly associated with the presence of GDM and the multivariate regression analysis further revealed that EAT thickness (OR = 2.87, 95% CI: 2.49–3.31) was significantly associated with the presence of GDM (*p* < 0.001) [59].

Although more substantial studies have yet to validate serum biomarkers as GDM predictors, the current findings are encouraging. For example, a study published by Zhao et al. in 2017 [60] describes the potential of five biological markers, respectively pentraxin 3, placental protein 13, myostatin, soluble FMS-like tyrosine kinase-1 and follistatin which presented detection rates of 94.9%, 92.3%, 92.3%, 94.9% and 92.3% with a Spe of 80%, when measured in the second trimester, between 16 and 20 weeks of gestation. Another article published by Lorenzo-Almorós et al. [61] in 2019 describes the possibility of predicting GDM in the first trimester based on the dynamics of specific plasmatic markers: decrease of plasmatic adiponectin and sex hormone-binding globulin (SHBG) combined with increased plasmatic levels of ficolin-3, afamin, retinol-binding protein 4 and specific micro-RNAs (miR), miR16-5p, miR-20a-5p and miR-17-5p. It is also hypothesized that VAT may predict GDM by regulating the miRNA-148 family of adipose-derived exosomes [62]. Another example is pregnancy-associated plasma protein A (PAPP-A), a study reporting that a low value was strongly associated with GDM, and lower values were found in women with GDM needing insulin therapy [8]. Nanda et al. also showed that in screening for GDM at 11–13 gestation weeks by maternal characteristics in which maternal age, BMI, racial origin, previous history of GDM and macrosomic neonate were significant independent predictors of future GDM, the detection rate was 61.6% at a false-positive rate of 20% and the detection increased to 74.1% by the addition of adiponectin and SHBG which were also lower in their study in the GDM group [63].

**Figure 2:**
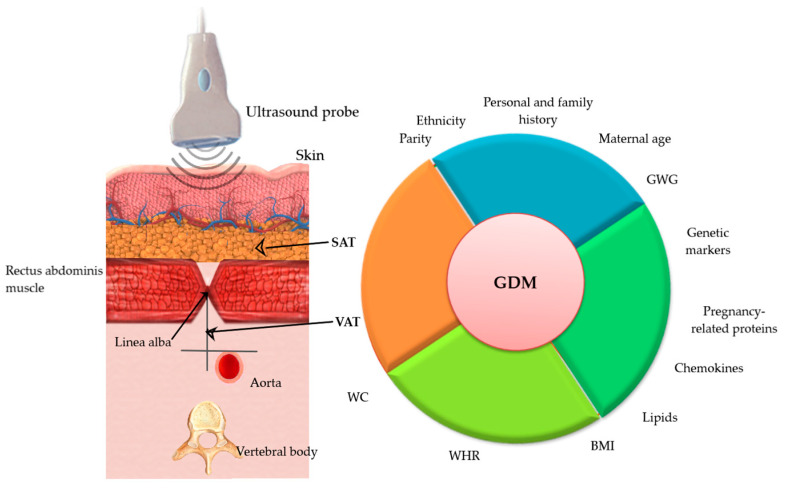
Ultrasound (US) measurement of adipose tissue and Parameters associated with GDM: anthropometric, genetic, biological and ultrasonographic. GDM-gestational diabetes mellitus, VAT-visceral adipose tissue thickness measured by US; SAT—subcutaneous adipose thickness measured by US, WC-waist circumference, WHR-waist-to-hip ratio, BMI-body mass index, GWG-gestational weight gain.

Therefore, a predictive model comprising risk factors, anthropometric variables, US-measured adipose thickness, especially VAT and biochemical markers could have a high predictive value of GDM as shown in Figure 2. However, more studies are needed in this aspect.

### 4.6. Possible Confounders

Most measurements were performed in the first trimester of pregnancy, between 11 gestational weeks and 13 gestational weeks and six days, aiming for a combined screening for fetal aneuploidies, pregnancy dating or determining fetal nuchal thickness. More studies are needed to determine if this is the optimal time to evaluate maternal adipose tissue. For instance, Alves et al. considered that a limitation of their study is that they enrolled women at the end of the first trimester of pregnancy, once some metabolic and habitus changes had already occurred. Although most studies performed adjustments for confounders, such as maternal age and BMI, it is challenging to balance gestational weight gain and other risk factors for GDM such as personal or family history. Despite the possible measurement error due to inter-operator variation, the US assessment technique also had some heterogeneity between studies and reported different cut-off values. The small sample of subjects may need to offer more statistical power to some studies.

Last but not least, the need for a gold standard to confidently identify GDM could be a limitation to any study assessing a new diagnostic or predictive tool. In addition, there are questions regarding the reliability of OGTT, as it involves a supra-physiological glucose load unrelated to body weight or dietary intake. Finally, although it is unpleasant, expensive and time-consuming, OGTT has poor reproducibility [64].

## 5. Conclusions

Pregnant women classified at an early stage as high risk for GDM may benefit from close monitoring and the introduction of preventive strategies with the primary objective of reducing the burden of DM in pregnancy and later life. US-measured VAT during the first trimester of pregnancy seems a practical and inexpensive screening approach that can predict GDM development later in pregnancy either by itself alone or can increase the prediction power when used in conjunction with other clinical and biological parameters. However, standardized procedures including VAT measurement technique, cut-off values, time of assessment and universally accepted diagnostic criteria for GDM are needed to validate this screening strategy through more large-scale trials.

## Figures and Tables

**Table 1:** Study main characteristics and evaluated factors.

Author and Year	Study Design	Country	No of Patients	Diagnostic Criteria	GW of Assessment	US Techinique	GDM Incidence	Results	Adjusting Factors
Alves et al., 2020 [7]	prospective cohort study	Brazil	518	IADPSG	<20 GW Mean 14.4 GW	Armellini et al., slightly modified by Martin et al.	87 (16.8%)	increased risk of GDM in relation to VAT in early pregnancy, similar after adjusting for BMI. OR 2.00, 95% CI 1.61 to 2.50	Age and pregestational BMI
D’Ambrosi et al., 2020 [29]	single-center study	Italy	295	IADPSG	11^2/7^–13^6/7^ GW	Suzuki et al.	57 (19.32%) 1st trimester (*n* = 29) 2nd trimester (*n =* 28)	Signifficant association for VAT and for 1st and 2nd trimester GDM	maternal age, BMI at 12 GW, GWG at 12 GW, parity and family history of DM
Bourdages et al., 2018 [30]	planned sub-cohort study of a large prospective cohort study	Canada	1048	ACOG	11^0/7^ to 14^0/7^ weeks	Armellini et al. Martin et al.	61 (5.8%) developed GDM, of which 36 (3.4%) insulin-requiring GDM.	VAT associated with subsequent GDM (AUC 0.65, 95% CI 0.58–0.73)	maternal age and BMI
Thaware et al., 2019 [28]	prospective observational study	United Kingdom	80	IADPSG/WHO 2013 criteria	9–18 GW	Armellini et al., slightly modified by Martin et al.	15 (19%)	VAT was associated with greater GDM risk 2.09 (95% CI 1.06–4.12; *p =* 0.03) for 1-SD increase	age, parity, years in education and pre-pregnancy BMI
Rocha et al., 2020 [25]	prospective cohort study	Brazil	133	IADPSG	≤20 GW	Armel-lini et al.	8 (13.5%)	Strong association between VAT at a 45 mm treshold andGDM. Crude and aOR for GDM were 13.4 (95% CI 2.9–61.1) and 8.9 (95% CI 1.9–42.2)	maternal age and pre-gravid BMI
De Souza et al., 2016 [33]	prospective cohort study	Canada	485	IADPSG	11–14 GW	Martin et al.	45 (9.27%) (9.3%, 95% CI 7.0–12.2)	The highest quartile of VAT (aOR 3.1, 95% CI 1.1–9.5) was associated with the composite outcome of GDM, IFG or IGT 3.4, 95% CI; 1.5–8.3 for IFG	maternal age, ethnicity, family history of type 2 DM, BMI at 11–14 GW and change in BMI from 11–14 to 24–28 GW
Gur et al., 2014 [34]	prospective cohort study	Turkey	94	FBG > 105 mg/dL 1 h glucose >190 mg/dL 2 h glucose > 165 mg/dL 3 h glucose > 145 mg/dL - 1 value above the cutoffs = IGT - 2 values above the cutoffs = GDM	4–14 GW	Martin et al.	IGT 6 (6.3%) GDM 10 (10.2%) MS 9 (9.5%)	VAT was significantly higher in the GDM group (*p =* 0.04) VAT in early pregnancy correlated with hyperglycemia, dyslipidemia, high diastolic BP, and IR. VAT was a more sensitive predictor of GDM than WC and BMI.	diastolic BP, TG, FBG, insulin level, HOMA-IR, HDL, BMI, WC, age, DM family history and GDM history
De Souza et al., 2016 [35]	prospective cohort study	Canada	476	CDA	11–14 GW	De Souza et al., 2014	50 (10.5%) developed the composite of IFG, IGT or GDM	Maternal hepatic fat and abdominal adiposity (VAT, TAT) may independently predict disglycemia and GDM in mid-pregnancy.	maternal age, ethnicity, 1st degree relative with type 2 DM, BMI at 11–14 GW and change in BMI from 11–14 to 24–28 GW
Saif Elnasr et al., 2021 [36]	observational study	Egypt	83	ADA	11–14 GW	Muller et al.	12 (14.45%) GDM	- positive relationship between VAT and HOMA-IR. - negative relationship between VAT and insulin Sen.	HOMA-IR, ISI and BMI
Tunc et al., 2022 [37]	observationalstudy	Turkey	100	IADPSG	11–14 GW	Martin et al.	12 (12%) GDM	The most significant risk factor for the prediction of GDM was VAT (OR = 33.2, 95% CI = 7.395–149.046, *p* < 0.001). Other signifficant predictors were SAT, TAT, a pre-gestational BMI > 30 kg/m^2^	maternal age, parity, GW at recruitment FPG, plasma insulin, HOMA-IR, HDL, LDL, VLDL, TG, systolic BP diastolic BP, BMI, Pre-gestational-BMI and body weight and GWG.
Gupta et al., 2022 [38]	cohort study	India	190	IADPSG	11–14 GW	Muller et al.	98 (51.57%)	There was a significant association between SAT, VAT and BMI and occurrence of GDM, *p* < 0.001	age, gestational age, thyroid stimulating hormone, SAT and TAT.

GW—gestational week; WHO—World Health Organization; IADPSG—The International Association of Diabetes and Pregnancy Study Groups; ACOG—American College of Obstetricians and Gynecologists; ADA—American Diabetes Association; CDA—Canadian Diabetes Association; DM—diabetes mellitus; GDM—gestational DM; US—ultrasound; BMI—body mass index; SD—standard deviation; aOR—adjusted odds ratio; OR—odds ratio; CI—confidence interval; VAT—visceral adipose tissue; SAT—subcutaneous adipose tissue; TAT—total adipose tissue; FBG—fasting blood glucose; IGT—impaired glucose tolerance; IFG—impaired fasting glucose; WC—waist circumference; TG—triglycerides; HDL—HDL cholesterol; LDL—LDL cholesterol; VLDL—VLDL cholesterol; MS—metabolic syndrome (diagnosed when three or more risk factors were present, as by the International Diabetes Federation 2005 criteria: BMI ≥ 30 kg/m^2^, TG ≥ 150 mg/dL, HDL ≥ 50 mg/dL, FBG ≥ 100 mg/dL and blood pressure ≥130/≥85 mmHg); ISI—insulin sensitivity index; GWG—gestational weight gain; BP—blood pressure; WHR—waist/hip ratio.

**Table 2:** Results of the included studies.

Author and Year	Maternal Mean Age (Years + SD)	VAT Depth (mm + SD)	SAT Depth (Value/NR)	Other US Parameters (Value/NR)	Anthropometric Indices (Mean + SD)	Detailed Results	Prediction Power/Special Considerations
Alves et al., 2020 [7]	GDM group 27.5 ± 5.8 Non-GDM group 26.0 ± 5.7	54.4 ± 12.7 GDM group 63 ± 13 Non-GDM group 52 ± 11	NR	NR	GDM group - Pre-pregnancy BMI = 25.4 ± 4.6 kg/m^2^ Non-GDM group - Pre-pregnancy BMI = 24.4 ± 4.5 kg/m^2^	VAT and FPG (r = 0.179, 95% CI 0.094–0.261; *p* < 0.001) VAT and OGTT 1 h glucose (r = 0.238, 95% CI 0.154–0.319; *p* < 0.001) VAT and OGTT 2 h glucose (r = 0.221, 95% CI 0.136 to 0.303; *p* < 0.001) VAT-GDM—OR 2.00, 95% CI 1.61–2.50, *p =* 0.001 VAT (0.70 95% CI 0.63–0.75) vs. pre-pregnancy BMI (0.57 95% CI 0.50–0.64) (*p* < 0.0001)	VAT was more predictive for GDM than pre-pregnancy BMI. Optimal VAT cut-off for maximized Youden’s index was 5.1 cm, and a 1 cm increase in VAT led to unadjusted OR for developing GDM of 1.99 (95% CI 1.59–2.46)
D’Ambrosi et al., 2020 [29]	GDM group (1st trimester) 33.4 ± 4.3 GDM group (2nd trimester) 33.3 ± 4.1 Non-GDM group 33.0 ± 4.3	GDM group (1st trimester) 99 ± 44 GDM group (2nd trimester) 105 ± 53 Non-GDM group 72 ± 35	GDM group (1st trimester) 128 ± 65 GDM group (2nd trimester) 111 ± 46 Non-GDM group 98 ± 49	NR	GDM group (1st trimester) - BMI at 12 GW 24.6 (4.8) kg/m^2^ - GWG at 12 GW 2.0 (1.1) kg GDM group (2nd trimester) - BMI at 12 GW—24.8 (4.8) kg/m^2^ - GWG at 12 GW 1.6 (1.0) kg Non-GDM group - BMI at 12 GW 22.2 (3.9) kg/m^2^ - GWG at 12 GW 1.7 (1.1) kg	VAT *p =* 0.01 (Multivariate analysis) BMI *p* < 0.01 (Univariate analysis) 1st trimester GDM OR = 1.15, 95% CI 1.02–1.29 and 2nd trimester GDM OR = 1.19, 95% CI 1.05–1.34	In the multivariate analysis, only VAT was significantly associated with the risk of GDM. No further association was observed in the multivariate analysis
Bourdages et al., 2018 [30]	Insulin-requiring GDM 30.4 ± 4.6 GDM—no insulin 29.3 ± 3.2 No GDM 28.9 ± 4.1	NR	NR	NR	Insulin-requiring GDM - Maternal weight 81.3 (21.1) kg - BMI 30.0 (7.4) kg/m^2^ GDM—no insulin - Maternal weight 69.3 (17.9) kg - BMI 26.7 (6.7) (kg/m^2^) No GDM - Maternal weight 66.7 (14.0) kg - BMI 24.8 (5.0) kg/m^2^	GDM - SAT (AUC 0.66, 95% CI 0.59–0.73) - VAT (AUC 0.65, 95% CI 0.58–0.73) - TAT (AUC 0.68, 95% CI 0.61–0.76) Insulin-requiring GDM - SAT (AUC 0.70, 95% CI 0.61–0.79) - VAT (AUC 0.73, 95% CI 0.65–0.82) - TAT (AUC 0.76, 95% CI 0.67–0.84)	In logistic regression, at a false-positive rate of 10%, the detection rates for insulin-requiring GDM were 19% (95% CI 8–36) for maternal age ≥35 years, 31% (95% CI 16–48) for a BMI ≥31.6 kg/m^2^ and 31% (95% CI 16–48) for TAT ≥61 mm, up to 42% (95% CI 26–59) in a model including all 3 measures.
Thaware et al., 2019 [28]	N	43.6 ± 13.1	22.4 ± 10.1	NR	NR	VAT (OR 2.09, 95% CI 1.06–4.12; *p =* 0.03) SAT (OR 0.62; 95% CI 0.27–1.44; *p =* 0.27)	Increasing VAT, but not SAT, was associated with greater GDM risk after adjusting for confounding factors. VAT ≥ 42.7 mm had greater Sen and similar Spe compared with current NICE criteria for GDM.
Rocha et al., 2020 [25]	26 ± 6.2	Pre-pregnancy BMI < 25.0 kg/m^2^ 37.0 ± 12.5 Pre-pregnancy BMI 25.0–30.0 kg/m^2^ 44.0 ± 11.2 Pre-pregnancy BMI > 30 kg/m^2^ 53.1 ± 14.8	NR	NR	NR	VAT 45 mm threshold aOR = 8.9 (1.9–42.2) for pre-gravid Obese and threshold of 45 mm; aOR = 6.1 (0.7–55.3) for Pre-gravid Non-obese and threshold of 45 mm	Significantly different VAT means between GDM (VAT = 55.4 ± 11.4 mm) and non-GDM (VAT = 42.5 ± 11.4 mm).
De Souza et al., 2016 [33]	32.9 ± 4.8	41 ± 17	19 ± 8	TAT 59 ± 21	BMI at 11–14 GW 25.1 ± 5.1 kg/m^2^	SAT (highest quartile) - Traditional criteria (aOR 1.8, 95% 0.70–4.8) - CDA criteria (aOR 1.5, 95% 0.56–4.5) - IADPSG criteria (aOR 2.0, 95% 0.95–4.5) VAT- highest quartile - Traditional criteria (aOR 3.1, 95% 1.1–9.5) - CDA criteria (aOR 4.2, 95% 1.4–14.2) - IADPSG criteria (aOR 3.4, 95% 1.5–8.0) TAT- highest quartile - Traditional criteria (aOR 2.7, 95% 1.1–7.8) - CDA criteria (aOR 3.0, 95% 1.1–8.9) - IADPSG criteria (aOR 3.4, 95% 1.6–7.7)	The highest quartile of VAT and TAT were each associated with the composite outcome (GDM, IFG, IGT).
Gur et al., 2014 [34]	Normal glucose metabolism 47.5 IGT 53.9 GDM 43.4	Normal glucose metabolism VAT max = 44.9 IGT VAT max = 48.1 GDM VAT max = 67.2	Normal glucose metabolismSAT min = 44.9 IGTSAT min = 48.7 GDM SAT min = 66.7	NR	Normal glucose metabolism - BMI 45.1 kg/m^2^ - WC 45.2 cm IGT - BMI 43.1 kg/m^2^ - WC 46.9 cm GDM - BMI 68.1 kg/m^2^ - WC 65.3 cm	VAT max *p =* 0.04 SAT min *p =* 0.06	optimal cutoff points predicting disglycemia were VAT max = 19.5 mm (AUC = 0.66, *p =* 0.043), WC = 103.5 cm (AUC = 0.64, *p =* 0.079), and BMI = 34.5 (AUC = 0.64, *p =* 0.069).
De Souza et al., 2016 [35]	32.9 ± 4.8	NR	NR	hepatic fat NR TAT NR	- BMI at 11–14 GW25.1 ± 5.1 - change in BMI from 11–14 to 24–28 GW 2.6 ± 1.8	- hepatic fat + Q4 of VAT (aOR 6.5, 95% CI 2.3–18.5 - hepatic fat absent + Q4 of VAT (aOR 2.3, 95% CI 1.0–5.4. - hepatic fat + Q4 of TAT (aOR 7.8, 95% CI 2.8–21.7)	Association was independently of maternal age, ethnicity, family history of type 2 DM or maternal BMI.
Saif Elnasr et al., 2021 [36]	26.8	GDM group 58.5 ± 4.7 Non-GDM group 23 ± 6	GDM group 18 ± 5.7 Non-GDM group 16.6 ± 5.9	NA	GDM group - BMI kg/m^2^ 33.92 ± 8.16 - HOMA-IR 0.416 ± 0.03 - ISI 0.79 ± 0.10 Non-GDM group - BMI kg/m^2^ 23.32 ± 1.90 - HOMA-IR 0.254 ± 0.050 - ISI 0.18 ± 0.06	GDM vs non-GDM: - VAT *p =* 0.001 - SAT *p =* 0.451 - HOMA-IR *p =* 0.001 - ISI *p =* 0.001	No significant relationship between SAT and HOMA-IR.
Tunc et al., 2022 [37]	GDM group 29.5 ± 6.29 Non-GDM group 27.31 ± 5.38	GDM group 24.75 ± 10.34 Non-GDM group 16.68 ± 6.73	GDM group 26.33 ± 5.33 Non-GDM group 17.68 ± 4.86	NR	GDM group - BMI 36.17 ± 5.36 kg/m^2^ - Pre-gestational BMI 34.18 ± 5.39 kg/m^2^ - Pre-gestational body weight 85.5 ± 11.33 kg - GWG 6.5 ± 4.85 kg Non-GDM group - BMI 26.97 ± 4.89 kg/m^2^ - Pre-gestational BMI 25.66 ± 4.67 kg/m^2^ - Pre-gestational body weight 65.23 ± 12.31 kg - GWG 4.41 ± 3.02 kg	GDM prediction: - Pre-gestational - BMI > 30 kg/m^2^ (Sen = 75.0%, Spe = 78.41%). - BMI in the 1st trimester (Sen = 75.0%, Spe = 54.55%). - VAT > 18 mm (Sen = 75.0%, Spe = 78.18%). - SAT > 25 mm (Sen = 66.67%, Spe = 85.45%). - TAT > 44 mm (Sen = 75.0%, Spe = 81.82%).	The mean VAT and TAT were significantly higher in the GDM group *p* < 0.001
Gupta et al., 2022 [38]	23.24 ± 2	NR	NR	TAT	BMI kg/m^2^ 20.67	Association with GDM - VAT *p* < 0.001 - SAT *p* < 0.001 - TAT *p* < 0.001 - BMI *p =* 0.001 - Family History of DM, *p =* 0.012 GDM prevalence increased with VAT, SAT and TAT increase.	A logistic regression utilizing age, gestational age, TSH, VAT, SAT, TAT as predictors was statiscally significant (chi square = 56.311, df = 8, *p =* 0.001)

IADPSG—The International Association of Diabetes and Pregnancy Study Groups; NICE-National Institute of Health and Care Excellence; NR—not reported; VAT—visceral adipose tissue; SAT—subcutaneous adipose tissue; TAT—total adipose tissue; VAD—visceral adipose depth; SD—standard deviation; OR—odds ratio; aOR—adjusted odds ratio; CI—confidence interval; df-degrees of freedom; DM—diabetes mellitus; GDM—gestational DM; US—ultrasound; BMI—body mass index; WC—waist circumference; WHR—waist/hip ratio; GWG—Gestational weight gain; GW—gestational weeks; FBG—fasting blood glucose; IGT—impaired glucose tolerance; TSH—thyroid stimulating hormone; Sen—sensitivity; Spe—specificity.

## Data Availability

Not applicable. **Aknowledgment:** Publication of this paper was supported by the University of Medicine and Pharmacy Carol Davila, through the institutional program Publish not Perish.

## References

[1] International Diabetes Federation Gestational Diabetes. https://www.idf.org/our-activities/care-prevention/gdm#:~:text=There%20were%20an%20estimated%20223,were%20due%20to%20gestational%20diabetes.

[2] Gregory E.C., Ely D.M. (2022). Trends and Characteristics in Gestational Diabetes: United States, 2016–2020. Natl. Vital Stat. Rep..

[3] Bilous R.W., Jacklin P.B., Maresh M.J., Sacks D.A. (2021). Resolving the Gestational Diabetes Diagnosis Conundrum: The Need for a Randomized Controlled Trial of Treatment. Diabetes Care.

[4] American Diabetes Association Professional Practice Committee (2022). 2. Classification and Diagnosis of Diabetes: Standards of Medical Care in Diabetes—2022. Diabetes Care.

[5] Wang H., Li N., Chivese T., Werfalli M., Sun H., Yuen L., Ambrosius Hoegfeldt C., Powe C.E., Immanuel J., Karuranga S. (2022). IDF Diabetes Atlas: Estimation of Global and Regional Gestational Diabetes Mellitus Prevalence for 2021 by International Association of Diabetes in Pregnancy Study Group’s Criteria. Diabetes Res. Clin. Pract..

[6] Waugh N., Pearson D., Royle P. (2010). Screening for hyperglycaemia in pregnancy: Consensus and controversy. Best Pract. Res. Clin. Endocrinol. Metab..

[7] Alves J.G., Souza A., Figueiroa J.N., de Araújo C., Guimarães A., Ray J.G. (2020). Visceral Adipose Tissue Depth in Early Pregnancy and Gestational Diabetes Mellitus—A Cohort Study. Sci. Rep..

[8] Lovati E., Beneventi F., Simonetta M., Laneri M., Quarleri L., Scudeller L., Albonico G., Locatelli E., Cavagnoli C., Tinelli C. (2013). Gestational diabetes mellitus: Including serum pregnancy-associated plasma protein-A testing in the clinical management of primiparous women? A case-control study. Diabetes Res. Clin. Pract..

[9] Sandu C., Bica C., Salmen T., Stoica R., Bohiltea R., Gherghiceanu F., Pacu I., Stefan S., Serafinceanu C., Stoian A.P. (2021). Gestational diabetes—Modern management and therapeutic approach (Review). Exp. Ther. Med..

[10] Bhattacharya S., Nagendra L., Krishnamurthy A., Lakhani O.J., Kapoor N., Kalra B., Kalra S. (2021). Early Gestational Diabetes Mellitus: Diagnostic Strategies and Clinical Implications. Med. Sci..

[11] Hedderson M.M., Williams M.A., Holt V.L., Weiss N.S., Ferrara A. (2008). Body mass index and weight gain prior to pregnancy and risk of gestational diabetes mellitus. Am. J. Obstet. Gynecol..

[12] Kim C., Liu T., Valdez R., Beckles G.L. (2009). Does frank diabetes in first-degree relatives of a pregnant woman affect the likelihood of her developing gestational diabetes mellitus or nongestational diabetes?. Am. J. Obstet. Gynecol..

[13] Zhang C., Ning Y. (2011). Effect of dietary and lifestyle factors on the risk of gestational diabetes: Review of epidemiologic evidence. Am. J. Clin. Nutr..

[14] Gibson K.S., Waters T.P., Catalano P.M. (2012). Maternal weight gain in women who develop gestational diabetes mellitus. Obstet. Gynecol..

[15] Hedderson M.M., Gunderson E.P., Ferrara A. (2010). Gestational weight gain and risk of gestational diabetes mellitus. Obstet. Gynecol..

[16] Getahun D., Fassett M.J., Jacobsen S.J. (2010). Gestational diabetes: Risk of recurrence in subsequent pregnancies. Am. J. Obstet. Gynecol..

[17] Zilberlicht A., Feferkorn I., Younes G., Damti A., Auslender R., Riskin-Mashiah S. (2016). The mutual effect of pregestational body mass index, maternal hyperglycemia and gestational weight gain on adverse pregnancy outcomes. Gynecol. Endocrinol..

[18] Metzger B.E., Lowe L.P., Dyer A.R., Trimble E.R., Chaovarindr U., Coustan D.R., Hadden D.R., McCance D.R., Hod M., HAPO Study Cooperative Research Group (2008). Hyperglycemia and adverse pregnancy outcomes. N. Engl. J. Med..

[19] Bobirca A., Bobirca F., Ancuta I.C., Mihai C., Tataru C., Comsa M., Bojinca M., Micu A., Musetescu C., Ancuta V. (2017). Pregnancy in rheumatoid arthritis—A Romanian cohort. Ann. Rheum. Dis..

[20] Bobirca A., Ancuta I., Bojincă M., Stoica V., Ceaușu I., Toader O., Micu M., Ancuta C., Mușetescu A., Bobirca F. Risk factors of adverse pregnancy outcome in Romanian rheumatoid arthritis patients. Proceedings of the 4th Congress of the Romanian Society for Minimal Invasive Surgery in Ginecology/Annual Days of the National Institute for Mother and Child Health Alessandrescu-Rusescu.

[21] Torloni M.R., Betrán A.P., Horta B.L., Nakamura M.U., Atallah A.N., Moron A.F., Valente O. (2009). Pre-pregnancy BMI and the risk of gestational diabetes: A systematic review of the literature with meta-analysis. Obes. Rev..

[22] Madhavan A., Beena Kumari R., Sanal M.G. (2008). A pilot study on the usefulness of body mass index and waist hip ratio as a predictive tool for gestational diabetes in Asian Indians. Gynecol. Endocrinol..

[23] Basraon S.K., Mele L., Myatt L., Roberts J.M., Hauth J.C., Leveno K.J., Varner M.W., Wapner R.J., Thorp J.M., Peaceman A.M. (2016). Relationship of Early Pregnancy Waist-to-Hip Ratio versus Body Mass Index with Gestational Diabetes Mellitus and Insulin Resistance. Am. J. Perinatol..

[24] Sina M., Hoy W.E., Callaway L., Wang Z. (2015). The associations of anthropometric measurements with subsequent gestational diabetes in Aboriginal women. Obes. Res. Clin. Pract..

[25] Rocha A., Bernardi J.R., Matos S., Kretzer D.C., Schöffel A.C., Goldani M.Z., de Azevedo Magalhães J.A. (2020). Maternal visceral adipose tissue during the first half of pregnancy predicts gestational diabetes at the time of delivery—A cohort study. PLoS ONE..

[26] Suresh A., Liu A., Poulton A., Quinton A., Amer Z., Mongelli M., Martin A., Benzie R., Peek M., Nanan R. (2012). Comparison of maternal abdominal subcutaneous fat thickness and body mass index as markers for pregnancy outcomes: A stratified cohort study. Aust. N. Z. J. Obstet. Gynaecol..

[27] Pontual A.C., Figueiroa J.N., De Souza L.R., Ray J.G., Alves J.G. (2016). Visceral Adiposity in the First Half of Pregnancy in Association with Glucose, Lipid and Insulin Profiles in Later Pregnancy: A Cohort Study. Matern. Child Health J..

[28] Thaware P.K., Patterson C.C., Young I.S., Casey C., McCance D.R. (2019). Clinical utility of ultrasonography-measured visceral adipose tissue depth as a tool in early pregnancy screening for gestational diabetes: A proof-of-concept study. Diabet. Med..

[29] D’Ambrosi F., Rossi G., Soldavini C.M., Di Maso M., Carbone I.F., Cetera G.E., Colosi E., Ferrazzi E. (2020). Ultrasound assessment of maternal adipose tissue during 1st trimester screening for aneuploidies and risk of developing gestational diabetes. Acta Obstet. Gynecol. Scand..

[30] Bourdages M., Demers M.É., Dubé S., Gasse C., Girard M., Boutin A., Ray J.G., Bujold E., Demers S. (2018). First-Trimester Abdominal Adipose Tissue Thickness to Predict Gestational Diabetes. J. Obstet. Gynaecol. Can..

[31] Aydın G.A., Özsoy H.G.T., Akdur P.Ö., Özgen G. (2021). The predictive value of first-trimester anthropo-metric and ultrasonographic adipose tissue measurements in gestational diabetes mellitus. J. Obstet. Gynaecol. Res..

[32] Wells G.A., Shea B., O’Connell D., Peterson J., Welch V., Losos M., Tugwell P. (2011). The Newcastle-Ottawa Scale (N.O.S.) for Assessing the Quality of Nonrandomized Studies in Meta-Analyses.

[33] De Souza L.R., Berger H., Retnakaran R., Maguire J.L., Nathens A.B., Connelly P.W., Ray J.G. (2016). First-Trimester Maternal Abdominal Adiposity Predicts Dysglycemia and Gestational Diabetes Mellitus in Midpregnancy. Diabetes Care.

[34] Gur E.B., Ince O., Turan G.A., Karadeniz M., Tatar S., Celik E., Yalcin M., Guclu S. (2014). Ultrasonographic visceral fat thickness in the first trimester can predict metabolic syndrome and gestational diabetes mellitus. Endocrine.

[35] De Souza L.R., Berger H., Retnakaran R., Vlachou P.A., Maguire J.L., Nathens A.B., Connelly P.W., Ray J.G. (2016). Hepatic fat and abdominal adiposity in early pregnancy together predict impaired glucose homeostasis in mid-pregnancy. Nutr. Diabetes.

[36] SaifElnasr I., Ammar H. (2021). Ultrasound markers for prediction of gestational diabetes mellitus in early pregnancy in Egyptian women: Observational study. J. Matern.-Fetal Neonatal Med..

[37] Tunc S., Oglak S.C., Olmez F., Ozkose Z.G. (2022). The Value of First-trimester Maternal Abdominal Visceral Adipose Tissue Thickness in Predicting the Subsequent Development of Gestational Diabetes Mellitus. J. Coll. Physicians Surg. Pak..

[38] Gupta S., Gupta A., Swarnakar C.P., Rathore M., Beniwal R., Meena K., Simlot A., Gupta N. (2022). The Early Sonographic Prediction of Gestational Diabetes in Women from India. J. Diagn. Med. Sonogr..

[39] D’Ambrosi F., Crovetto F., Colosi E., Fabietti I., Carbone F., Tassis B., Motta S., Bulfoni A., Fedele L., Rossi G. (2018). Maternal Subcutaneous and Visceral Adipose Ultrasound Thickness in Women with Gestational Diabetes Mellitus at 24–28 Weeks’ Gestation. Fetal Diagn. Ther..

[40] Martin A.M., Berger H., Nisenbaum R., Lausman A.Y., MacGarvie S., Crerar C., Ray J.G. (2009). Abdominal visceral adiposity in the first trimester predicts glucose intolerance in later pregnancy. Diabetes Care.

[41] Rahnemaei F.A., Abdi F., Pakzad R., Sharami S.H., Mokhtari F., Kazemian E. (2022). Association of body composition in early pregnancy with gestational diabetes mellitus: A meta-analysis. PLoS ONE.

[42] Alwash S.M., McIntyre H.D., Mamun A. (2021). The association of general obesity, central obesity and visceral body fat with the risk of gestational diabetes mellitus: Evidence from a systematic review and meta-analysis. Obes. Res. Clin. Pract..

[43] Armellini F., Zamboni M., Rigo L., Todesco T., Bergamo-Andreis I.A., Procacci C., Bosello O. (1990). The contribution of sonography to the measurement of intra-abdominal fat. J. Clin. Ultrasound.

[44] Suzuki R., Watanabe S., Hirai Y., Akiyama K., Nishide T., Matsushima Y., Murayama H., Ohshima H., Shinomiya M., Shirai K. (1993). Abdominal wall fat index, estimated by ultrasonography, for assessment of the ratio of visceral fat to subcutaneous fat in the abdomen. Am. J. Med..

[45] Cremona A., Hayes K., O’Gorman C.S., Laighin C.N., Ismail K.I., Donnelly A.E., Hamilton J., Cotter A. (2019). Inter and intra-reliability of ultrasonography for the measurement of abdominal subcutaneous & visceral adipose tissue thickness at 12 weeks gestation. BMC Med. Imaging.

[46] Habibi N., Mousa A., Tay C.T., Khomami M.B., Patten R.K., Andraweera P.H., Wassie M., Vandersluys J., Aflatounian A., Bianco-Miotto T. (2022). Maternal metabolic factors and the association with gestational diabetes: A systematic review and meta-analysis. Diabetes Metab. Res. Rev..

[47] Bartha J.L., Marin-Segura P., Gonzalez- Gonzalez N.L., Wagner F., Aguilar-Diosdado M., Hervi-as-Vivancos B. (2007). Ultrasound evaluation of visceral fat and metabolic risk factors during early pregnancy. Obesity (Silver Spring).

[48] De Souza L.R., Kogan E., Berger H., Alves J.G., Lebovic G., Retnakaran R., Maguire J.L., Ray J.G. (2014). Abdominal adiposity and insulin resistance in early pregnancy. J. Obstet. Gynaecol. Can..

[49] Gutch M., Kumar S., Razi S.M., Gupta K.K., Gupta A. (2015). Assessment of insulin sensitivity/resistance. Indian J. Endocrinol. Metab..

[50] Lewandowski K., Głuchowska M., Garnysz K., Horzelski W., Grzesiak M., Lewiński A. (2022). High prevalence of early (1st trimester) gestational diabetes mellitus in Polish women is accompanied by marked insulin resistance—Comparison to PCOS model. Endokrynol. Pol..

[51] Swainson M.G., Batterham A.M., Tsakirides C., Rutherford Z.H., Hind K. (2017). Prediction of whole-body fat percentage and visceral adipose tissue mass from five anthropometric variables. PLoS ONE.

[52] Peña-Cano M.I., Valencia-Ortega J., Morales-Ávila E., Díaz-Velázquez M.F., Gómez-Díaz R., Saucedo R. (2022). Omentin-1 and its relationship with inflammatory factors in maternal plasma and visceral adipose tissue of women with gestational diabetes mellitus. J. Endocrinol. Investig..

[53] Rancourt R.C., Ott R., Ziska T., Schellong K., Melchior K., Henrich W., Plagemann A. (2020). Visceral Adipose Tissue Inflammatory Factors (TNF-Alpha, SOCS3) in Gestational Diabetes (GDM): Epigenetics as a Clue in GDM Pathophysiology. Int. J. Mol. Sci..

[54] Kelley D.E., Thaete F.L., Troost F., Huwe T., Goodpaster B.H. (2000). Subdivisions of subcutaneous abdominal adipose tissue and insulin resistance. Am. J. Physiol. Endocrinol. Metab..

[55] Yang S.H., Kim C., An H.S., An H., Lee J.S. (2017). Prediction of gestational diabetes mellitus in pregnant Korean women based on abdominal subcutaneous fat thickness as measured by ultrasonography. Diabetes Metab. J..

[56] Kennedy N.J., Peek M.J., Quinton A.E., Lanzarone V., Martin A., Benzie R., Nanan R. (2016). Maternal abdominal subcutaneous fat thickness as a predictor for adverse pregnancy outcome: A longitudinal cohort study. BJOG.

[57] Cooray S.D., Wijeyaratne L.A., Soldatos G., Allotey J., Boyle J.A., Teede H.J. (2020). The Unrealized Potential for Predicting Pregnancy Complications in Women with Gestational Diabetes: A Systematic Review and Critical Appraisal. Int. J. Environ. Res. Public Health.

[58] Kretzer D.C., Matos S., Von Diemen L., de Azevedo Magalhães J.A., Schöffel A.C., Goldani M.Z., da Silva Rocha A., Bernardi J.R. (2020). Anthropometrical measurements and maternal visceral fat during first half of pregnancy: A cross-sectional survey. BMC Pregnancy Childbirth.

[59] Liu J., Song G., Meng T., Zhao G. (2020). Epicardial adipose tissue thickness as a potential predictor of gestational diabetes mellitus: A prospective cohort study. BMC Cardiovasc. Disord..

[60] Zhao B., Han X., Meng Q., Luo Q. (2018). Early second trimester maternal serum markers in the prediction of gestational diabetes mellitus. J. Diabetes Investig..

[61] Lorenzo-Almorós A., Hang T., Peiró C., Soriano-Guillén L., Egido J., Tuñón J., Lorenzo Ó. (2019). Predictive and diagnostic biomarkers for gestational diabetes and its associated metabolic and cardiovascular diseases. Cardiovasc. Diabetol..

[62] Zhang Z., Xu Q., Chen Y., Sui L., Jiang L., Shen Q., Li M., Li G., Wang Q. (2021). The possible role of visceral fat in early pregnancy as a predictor of gestational diabetes mellitus by regulating adipose-derived exosomes miRNA-148 family: Protocol for a nested case-control study in a cohort study. BMC Pregnancy Childbirth.

[63] Nanda S., Savvidou M., Syngelaki A., Akolekar R., Nicolaides K.H. (2011). Prediction of gestational diabetes mellitus by maternal factors and biomarkers at 11 to 13 weeks. Prenat. Diagn..

[64] Di Filippo D., Wanniarachchi T., Wei D., Yang J.J., Mc Sweeney A., Havard A., Henry A., Welsh A. (2021). The diagnostic indicators of gestational diabetes mellitus from second trimester to birth: A systematic review. Clin. Diabetes Endocrinol..

